# Debt, Ownership, and Size: The Case of Hospitals in Poland

**DOI:** 10.3390/ijerph18094596

**Published:** 2021-04-26

**Authors:** Katarzyna Miszczyńska, Piotr Miszczyński

**Affiliations:** 1 Department of Public Finance, Faculty of Economics and Sociology, University of Lodz, 90-214 Lodz, Poland; 2 Department of Operations Research, Faculty of Economics and Sociology, University of Lodz, 90-214 Lodz, Poland; piotr.miszczynski@uni.lodz.pl

**Keywords:** financial condition, financial performance, indebtedness, health economics, healthcare

## Abstract

The goal of this study is to compare the financial performance of public hospitals according to ownership and size. The study covered public hospitals in Poland and covered two hospitals types depending on their founding authority, i.e., hospitals established and financed by the Marshal’s Office (Marshal hospitals) or the City Hall (poviat-commune hospitals). The study was based on an analysis of the hospitals’ financial situation (using debt and solvency ratios) and its relationship to the founding body and size. The verification of hypotheses was carried out using the Mann–Whitney U test. The results led to the conclusion that the vast majority of public hospitals are indebted, and their ownership structure does not affect their financial condition. The study did not confirm a significant relationship between size or ownership and the financial status of the hospital. The article aims to fill the research gap regarding the debt analysis between different types of public hospitals. It also presents a new research direction aimed at finding the factors that determine the difficult financial situation of public hospitals in Poland.

## 1. Introduction

Healthcare is a desirable good, and its inherent financing mechanism is key to achieving the macroeconomic goals of the healthcare system. These goals include controlling the level and dynamics of cost increases, and providing effective medical facilities, taking into account the quality and availability of healthcare services.

The healthcare system in Poland operates based on an insurance model in which the health insurance premiums of the insured finance the public sphere of healthcare. The most important element of that sort of financing is extra-budgetary funds, i.e., obligatory universal health insurance in the form of a premium (paid by each citizen, depending on income) [1]. Health insurance contributions are at the disposal of the National Health Fund, the state budget, and local government units. The funds from the extra-budgetary economy include earmarked funds, as well as subsidies from founding bodies. Private sector funding takes the form of (commercial) health insurance, household expenditure, or foundation funds. The last group includes funds from foreign aid.

Under the Act on healthcare entities [2], healthcare entities in Poland can be divided into enterprises and non-enterprises. The latter group includes hospitals organized in the form of independent public health care units (SPZOZ), budgetary units, or research institutes. Healthcare entities that are enterprises and non-enterprises differ from each other on many levels. The legal form of entities is extremely important from the point of view of how they function, as it involves both limitations and privileges for some entities.

Hospitals constitute the largest group of medical entities in Poland, and they vary in terms of status (public, private), ownership/founding body, financial objectives (for-profit, not-for-profit), size, or specialization (e.g., general, psychiatric) [3]. Those characteristics can significantly affect hospitals’ performance and financial standing [4]. Generally, hospitals should finance their growth with debt or revenue from the services they provide [5]. However, their funding sources for hospitals vary depending on their founding body, business profile, and whether they are public or private.

Public hospitals are the basic types of units that provide stationary and round-the-clock health services in Poland. They absorb most of the funds allocated to healthcare as that they are obliged to provide each patient not only with health services but also pharmaceuticals (including medical materials), rooms, and food that is appropriate to the patient’s state of health. Poland’s constitution obliges them to provide necessary assistance to every citizen in a life-threatening situation, regardless of their financial condition [6]. These benefits are financed from public funds and are provided free of charge, partially or fully paid. At the same time, patients pay for services financed from public funds at official prices and only in the event that it is provided for by separate regulations [7].

From the perspective of ownership structure and main founding body, public hospitals can be divided into university, Ministry, Marshal, and poviat-commune hospitals. Their activities are financed from the funds of the National Health Fund, public administration bodies (i.e., the Ministry of Health financing highly specialized procedures), local government units (in the field of financing health programs), and the European Union [6]. Thus, different sources of financing are possible depending on the type of hospital. For example, public hospitals in Poland that are not enterprises and whose founding bodies can be both local government units, universities or ministries receive funding [8]:

- from paid medical activities, i.e., under the provisions of the contract with the National Health Fund and contracts concluded directly with the Ministry of Health (highly specialized services);
- from a separate activity other than the provision of health services, if the statute provides for such an activity;
- from interest on deposits;
- from donations, bequests, inheritances, and public donations, also of foreign origin;
- to cover the negative financial result from the creating authority;
- from commissioned tasks, including health programs;
- from subsidies of the founding bodies.

Financing in the form of subsidies is classified in the literature as internal financing, while external sources of funding are usually connected with debt [5,9]. However, it should be emphasized that the founding units (in particular, the self-government units considered in the study) do not have full financial decision-making power in public hospitals and thus have limited influence on shaping their debt. Therefore, their financial situation may differ depending on the founding entity that the management adopted.

As was mentioned above, the healthcare sector has a special relationship with local government units as a result of the reform introduced in 1999, covering the following systems: administration, pension, education, and health protection [10]. The consequence of the administrative reform was the introduction of the three-stage new administrative division (communes, poviats, and voivodships). Its effect was the improvement of the efficiency of public funds management. Local government units took over the responsibility for the functioning of healthcare at the local and regional level, performing ownership functions in relation to poviat-commune and voivodeship hospitals, as well as many outpatient specialist care facilities [11]. Local government units acquired the rights to establish, liquidate, statute, supervise, and transform infrastructural healthcare facilities under their control. They are also responsible for financial management [10]. Establishing hospitals by local government units, as well as by government administration bodies, is to guarantee that these units will achieve the goals for which they were established [12].

Hospitals are a special type of enterprise that cannot be regarded as a typical market participant. This is due to the fact that hospitals are closely related to the National Health Fund (which guarantees them income) and are subordinate to their founding bodies [10]. Hospitals cover the costs of their operations and the negative financial result from their revenues. However, if this is impossible, the loss must be covered by the local government unit.

Due to numerous problems related to the functioning of hospitals, it seems necessary to work out and examine appropriate relationships aimed at assessing their situation. An important aspect also seems to be the assessment of the situation of hospitals depending on the specific founding body. Thus, the main goal of this article is to compare the financial performance of two types of hospitals: those founded by the Marshal’s Office (voivodeship hospitals) or the City Council (poviat-commune hospitals). The research is based on debt analysis performed in the perspective of the size and founding body of the hospital. Details regarding the methodology used and the characteristics of the research sample are presented in Section 2.

Based on the literature review and the current state of research, the following research questions were posed:

RQ 1: Are Marshal hospitals less indebted than poviat-commune ones?

RQ 2: Are large hospitals more indebted than medium-sized ones?

To answer these research questions, the following research hypotheses were formulated:

**Hypothesis** **1** **(H1).** **With regard to size, Marshal hospitals are less indebted than poviat-commune ones.**

**Hypothesis** **1a** **(H1a).** *Medium-sized Marshal hospitals are less indebted than poviat-commune ones.*

**Hypothesis** **1b** **(H1b).** *Large Marshal hospitals are less indebted than poviat-commune ones.*

**Hypothesis** **2** **(H2).** **With regard to ownership structure, large hospitals are more indebted than medium-sized ones.**

**Hypothesis** **2a** **(H2a).** *Large Marshal hospitals are more indebted than medium-sized ones.*

**Hypothesis** **2b** **(H2b).** *Large poviat-commune hospitals are more indebted than medium-sized ones.*

Despite the ongoing reforms of primary care in Poland, one of the most longstanding problems that need to be resolved is hospitals’ indebtedness [13]. Several studies conducted on highly indebted Polish hospitals underlined the existence of financial problems connected with ineffective management and ownership structures [14,15]. Bem et al. [4] showed that there is a positive relationship between the debt ratio and liquidity, as well as the profitability and liquidity ratio. Krzeczewski [10,16], who studied the impact of location on the financial condition of local government hospitals, confirmed that the founding body significantly affects the economic efficiency of the hospitals in the Lodz region. Miszczyńska [1] also confirmed the impact of the founding body on a hospital’s indebtedness in the Lodz region. The problem of ownership structure and its impact on the financial condition of hospitals has also been studied worldwide. Lee’s [17] financial analysis of Korean hospitals showed that national university hospitals were low indebted, and their management conditions seemed generally satisfactory. Ownership structure and financial performance has also been discussed by Bai and Anderson [18], Wheeler et al. [19], and Upadhyay and Smith [20]. The relationship between hospital size and its financial performance was studied by Kim [21], who noted that financial distress could have a detrimental influence hospital performance. The author also highlighted that hospital management needs to monitor potential financial distress effectively and know how it will respond depending on the severity of the circumstances. Bem et al. [22] showed a statistically significant relationship between annual income per bed and the level of liquidity. In other study, Bem et al. [5] also claimed that the size of a hospital affects, either positively or negatively, decisions made regarding new debt. Large Polish hospitals have better access to the financial market, and higher profitability ratios increase their chance of getting credit. By contrast, Michalski [23] and Gentry [9] showed that the financial position of public hospitals is too weak to be attractive to potential creditors, so they are supported by public donors. However, this statement was not widely confirmed in the literature on the subject [24].

The complexity of the medical and financial processes that take place in hospitals, along with the general constraints in the health sector (including a relatively low level of funding), mean there is a need for more research into debt analysis [4,13,25].

The vast majority of studies are usually based on the relationship between hospital financial performance, efficiency, and size [18,19,20,21,22,25,26,27,28,29]. At the same time, there are no studies that compare the indebtedness of different types of hospitals (measured by debt and solvency ratios) according to their ownership and size. Thus, there is a literature gap regarding comparisons between different types of hospital ownership (founding body) and debt. This is particularly important in the light of the frequently raised problem of hospital indebtedness in Poland [3,13]. Hence, this study is also aimed at filling this gap.

As there are no studies of this kind, the considerations we present mainly concern the financial analysis of hospitals with the main impact that their financial status has on their indebtedness. The changes that occur in hospitals’ performance due to their founding body are pointed out, as the founding body can influence the hospital’s financial management and, thus, its finances.

The structure of the paper is as follows. Section 1 presented the introduction and literature review. Section 2 presents the data and method. Section 3 presents and interprets the results of Mann–Whitney U tests on the relationships between indebtedness and size/founding body, while Section 4 the presents research discussion. The last section presents the conclusions.

## 2. Materials and Methods

The study is focused on hospitals that are public units; hence their primary source of financing is the National Health Fund, which is the main payer of the healthcare system in Poland. The study was conducted between 2013 and 2017. The data were obtained from the EMIS (Emerging Markets Information System) and Amadeus databases supplied by InfoCredit. As part of the study, data were collected on 321 public hospitals in Poland in terms of their founding body. The study was limited to 321 hospitals; as the remaining hospitals refused to disclose their financial statements, they were incomplete and therefore insufficient. It was also ensured that the regional (by voivodship) distribution of hospitals in the sample was statistically significantly consistent with the distribution (by voivodship) of all public healthcare system units. The statistics of the Mann–Whitney U test with a *p*-value of 0.85 (with a significance level of α = 0.05) indicated the statistical insignificance of differences between the distribution of the number of hospitals in the sample and in the population by voivodship [29].

The sample was to be representative of the study (see Table 1). To compare the two distributions, the χ^2^ test of homogeneity was carried out. The χ^2^ test was introduced according to the following notation:

(1) $$\chi^{2}=\sum_{j=1}^{k}\frac{{\left(O_{j}-E_{j}\right)}^{2}}{E_{j}}$$

where *O_j_* is the observed value of two nominal variables (in %) and *E_j_* is expected value of the two nominal variables (in %).

The tested hypothesis:

**In the sample, the distribution of hospitals according to the founding is consistent with the distribution for the entire population versus it is not consistent with the distribution for the entire population.**

**Figure 1:**
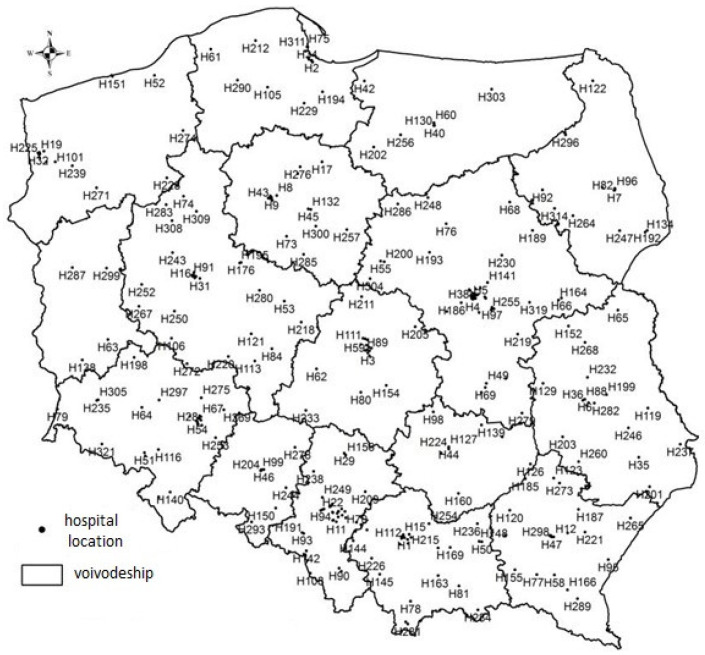
Location of the analyzed public hospitals. Note: H, conventional symbol of the hospital adopted for the purpose of the study.

Based on the test, there were no grounds to reject the null hypothesis. Thus, with 95% probability, it was found that the distribution of hospitals according to their founding body in the sample was consistent with the distribution of the entire population. Thus, the results of the analysis carried out in this study can be generalized to the entire population. Figure 1 shows the spatial distribution of the studied medical entities.

**Figure 2:**
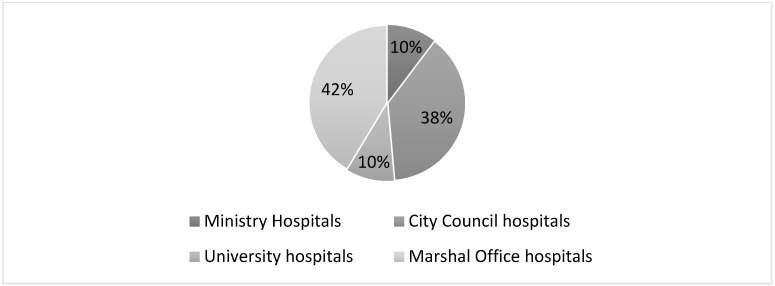
The structure of the analyzed hospitals according to the founding body.

The hospitals selected for the study constitute 34% of all healthcare entities in the country (out of 949 entities) and 58% of all public facilities (out of 580 entities) [3]. The structure of the analyzed units in terms of their founding body is shown in Figure 2. The largest group, i.e., approximately 42%, is made up of the Marshal’s Office and the City Council hospitals. Thus, the Marshal’s Office and the City Council hospitals are analyzed in the study.

The main aim of this article is to compare the financial performance of hospitals founded by the Marshal’s Office (voivodeship hospitals) or the City Council (poviat-commune hospitals). The authors tried to check whether the selected founding bodies of Polish public hospitals have any impact on their financial condition. Moreover, the relationship between the size of the hospital and its financial condition was also examined. The choice of variables used in the study was confirmed by an analysis of the literature on the subject (see Table 2).

According to the hospital size methodology, two approaches can be used. Using the size of the hospital is also confirmed in the literature and it fits with one of two approaches. Size can be measured by the number of hospital beds [1,6,10,18,20,21,23,24,26] or operating revenue and the value of total assets [33]. Generally, both approaches are used in the Polish healthcare sector studies. However, in this study, the authors chose to use the first approach. In the future, it is planned to check whether the analysis based on the second approach would bring statistically significantly different results.

For the purposes of the study, hospitals were divided into two groups based on their size. Thus, large and medium-sized hospitals were distinguished [25,31,32]. Such divisions are aimed at identifying the founding body’s influence on hospitals’ financial performance based on their ownership and size.

Below, we present how the size-group evaluation was prepared [4,42]:

- Medium-sized hospitals—number of hospital beds: 0–400;
- Large hospitals—number of beds: over 401.
- Using the methods of division described above, the following hospitals were selected for the study:
- 134 hospitals founded by the Marshal’s Office (Marshal hospitals), including 47 large hospitals and 87 medium-sized hospitals,
- 123 hospitals founded by the City Council (poviat-commune hospitals), including 34 large hospitals and 89 medium-sized hospitals.

Financial situation is measured by applying two ratios—debt (DT) and solvency (SLV)—in the period of 2013 and 2017. The ratio calculation method is presented below (see Table 3).

The scope of the study, which relates to the founding body, hospital size, and the financial situation (measured by SLV and DT), is supported by the in-depth literature analysis. The analysis of the functioning of hospitals from the perspective of debt ratios was intentional and resulted from the fact that the vast majority of public hospitals in Poland have struggled with indebtedness for many decades [13]. This indebtedness has a negative impact on everyday functioning and thus the quality of patient services. The subject literature presents a broad catalog of studies that touched on debt analysis, ownership structure, and size. However, in most cases, the inference in these spheres was independent. In the first step of the analysis, descriptive statistics of the analyzed variables were examined. The hypothesis of whether the indebtedness of Marshal hospitals differs significantly from the indebtedness of the poviat-commune hospitals was then verified. This process was done separately for each size. In the second part of the study, an analogous procedure was carried out for the ownership groups.

To test whether the variables are normally distributed in the analyzed period, selected tests were applied (a Lilliefors test based on Kolmogorov–Smirnov, Shapiro–Wilk, and χ^2^ tests). The test results revealed that the SLV ratio is normally distributed. However, DT had to be excluded from further research as it is not normally distributed in terms of medium-sized Marshal hospitals and large poviat-commune hospitals. Consequently, the non-parametric Mann–Whitney U test was used in the further analysis only in terms of the SLV ratio. In order to conduct the U Mann–Whitney test verification, dummy variables were created to group the hospitals into two categories (Marshal and poviat-commune).

A Z test was conducted to establish appropriate statistical significance levels according to the groupings. The following hypotheses are as follows:

**There are no differences in indebtedness between the analyzed groups.**
**There are differences in indebtedness between the analyzed groups.**

## 3. Results

The study aims to assess the indebtedness between Marshal and poviat-commune hospitals, and it also allows us to draw conclusions whether the sources and scope of hospital financing depend on the size or the founding body. The details concerning the descriptive statistics are presented in Table 4.

The normality of the analyzed variables was checked with the Lilliefors test (based on the Kolmogorov–Smirnov and Shapiro–Wilk tests) and c2 tests, respectively, depending on the number of sub-samples. As a result, most observations are not normally distributed. Exceptions are the DT ratio in all analyzed years for the medium-sized Marshal hospitals and large poviat-commune hospitals. That is why, as pointed out in the methodology section, those observations were excluded from further analysis, and to verify the hypotheses, we compared only SLV in all analyzed years.

In further analysis, the two stated research hypotheses were verified, first according to hospital size (Hypothesis 1) and then according to ownership/founding body (Hypothesis 2).

### 3.1. Large and Medium-Sized Hospitals

The verification was carried out using the Mann–Whitney U test; the research hypotheses are formulated as follows:

**There are no differences in indebtedness between the analyzed groups.**
**There occur differences in indebtedness between the analyzed groups.**

To verify the hypotheses stated in the methodology section, we employed a binary variable to divide the entire sample of medium-sized hospitals into two subsamples of Marshal (represented by 1) and poviat-commune (represented by 0) hospitals.

Table 5 presents some results of the Mann–Whitney U test. For the years 2013–2017, there is no reason to reject the null hypothesis (at the 0.05 significance level) regarding indebtedness. This means that indebtedness does not differ significantly between the groups in question. Thus, hospital ownership does not have any impact on indebtedness.

The procedure conducted for large hospitals was analogous to the procedure for medium-sized hospitals. Verification was carried out using the Mann–Whitney U test, and the sample of large hospitals was divided into two subsamples, of Marshal hospitals and poviat-commune hospitals.

### 3.2. Marshal and Poviat-Commune Hospitals

Analyzing the group of medium-sized and large Marshal hospitals, no significant differences occurred in the analyzed years. For the poviat-commune hospitals, in the whole analyzed period at the 0.05 significance level, there is also no reason to reject the null hypothesis regarding indebtedness (see Table 6). Thus, indebtedness does not differ significantly in the analyzed groups of hospitals.

## 4. Discussion

Despite the major changes in Polish healthcare over the last 30 years, Poland has free healthcare that is delivered through a publicly funded system. Thus, the vast majority of hospitals and public units are financed from public sources. A healthcare provision, which is built on accessibility, solidarity, equity, and quality, is the government’s responsibility [43,44,45]. As the literature review shows, the situation of the Polish healthcare sector is unsatisfactory [46,47]. The difficult financial situation of public hospitals is associated with growing debt, which negatively affects not only the development of healthcare but also the quality of medical services provided. This has created the need to determine what affects the difficult financial situation of healthcare units, which in turn has revealed several different economic and financial factors. From these factors, the solvency and debt ratios are most commonly used, which is why they were chosen for the analysis [48,49,50,51].

In the previous section, the statistics on the values of DT and SLV indicators were presented. The value of the solvency ratio, which determines the amount of external funds per unit of own fund, in the optimal range, suggested by the Ministry of Health, should fluctuate between 0.0 and 0.5 units. In 2013–2017, the average level of this indicator in the group of 321 hospitals studied was 0.64 and slightly exceeded the threshold set by the Ministry. However, the situation in individual groups was different. The average level of the solvency ratio for the Marshal’s hospitals was −2.3, and for poviat-commune hospitals −0.94. Nevertheless, there is a large spread of solvency in the sample, and most poviat and commune hospitals have a measure value from 0.1 to 3.75 units, and the marshal hospitals from 0.1 to 2.9. Moreover, among hospitals there are those with the solvency threshold of 188 (the maximum) and those for which this ratio obtained high negative values (the record low value of the solvency ratio was achieved in 2014, and it was −523 units). Negative values of the solvency ratio result from the negative value of equity, which in turn was influenced by the balance sheet item: loss from previous years or the lack of effective use of external financing. Over the analyzed years, the values of the index fluctuated constantly, and from 2016 they begin to increase. A high value of the ratio indicates the possibility of losing the entity’s ability to pay its liabilities.

Local government units face various problems related to running hospitals under their responsibility. Firstly, the main problem is the unclear role of local government units in the health care system. Secondly, the tasks and obligations of local government units have been defined very generally, and on the other hand, the introduced reform did not secure financial resources for local government units that are founding bodies of hospitals. Moreover, local government units are to be financially responsible for the operation of hospitals. However, they do not have complete control over their activities. Moving on to the results of the study, they are consistent with this situation and thus, comparing the analyzed sample of hospitals, the study showed that the founding body has no influence on the hospital’s financial situation. Despite the differences in the tools and the possibilities of supporting these two types of hospitals, there are no differences in their financial situation. Thus, Marshal hospitals, which are most often specialized hospitals and admit patients with more complex health conditions, are not characterized by worse financial situation. Nevertheless, they do not function better either. Moreover, no significant differences in the assessment of the financial situation were revealed, which would confirm the differences in the management of subordinate hospitals by the founding bodies. This means that the governing body cannot be treated as a factor affecting the financial efficiency of the hospital. These results are in contrast to those of Krzeczewski [10]. However, our study was based on a bigger representative sample, and it revealed that a hospital’s indebtedness depends on other economic and financial factors. The size of the hospital is also irrelevant. For both medium and large hospitals, the influence of the founding body on indebtedness is not statistically significant. This means that the additional financing options and support for hospital activities that are specific to each founding body are not as crucial for creating debt as, for example, the specificity of the unit [31] or its location in an urban or rural area [33]. We can also find confirmation for our results in the work of Baker et al. [52], where it was found that organizational outcomes are similar among hospital ownership types in relation to increasing administrative costs and overall medical efficiency. The obtained results are also consistent with those of Cygańska [53] and Bem et al. [22], who also did not notice a statistically significant relationship between the size of the hospital and the financial condition. These results are partially consistent with the study conducted by Antczak and Miszczyńska [3]. The research conducted by the authors clearly indicates that this trend does not have to apply to the entire country. The authors showed that there are differences in terms of the financial condition of hospitals from the perspective of different provinces. There are regions with hospitals that are very indebted and those whose debt ratio does not reach the ministerial level of 40% (0.4 units). In these regions, there are poviat-commune and voivodeship hospitals that are in a much worse situation and have greater financial problems, but also those that deal with the problem of debt much better than others (they do not make financial results dependent on one source of income, i.e., contracts with the National Health Fund, and are active in obtaining other revenues).

At the end of this study, one might raise the question if why Polish hospitals of different ownership types and sizes would have different levels of debt. Moreover, if the debt level is not a choice, what other processes would determine the resulting debt level for a hospital? The answers to these questions can be found in the principles of financing health services in Poland. As already mentioned, hospitals finance their medical activities mainly through the National Health Fund. Healthcare services are financed from public funds based on contracts regulated by the Civil Code. The rules for their conclusion are set out in the Act on health care services financed from public funds, and the orders of the President of the National Health Fund regulate the procedure for their conclusion. Based on the concluded contract, the National Health Fund only finances guaranteed services provided by the service provider selected in the course of the tender procedure. Agreements concluded between service providers and the National Health Fund are determined by procedures and the upper quantitative limit of services provided in a given financial year. Pursuant to the provisions of the 2015 ordinance of the Ministry of Health, the National Health Fund is obliged to finance services provided in the settlement period up to the amount of the Fund’s liability to the service provider specified in the contract for a given scope of services. However, there are some exceptions to these regulations, i.e., the so-called extra-limit benefits, which occur when a hospital admits a patient for treatment and has already used the entire contract concluded with the National Health Fund. Such situations arise in the case of life-saving procedures and childbirth among others. In such circumstances, the hospital cannot refuse to admit a patient who will generate costs not covered by the contract. Of course, the hospital may apply to the National Health Fund to refinance these funds, but the procedure is time-consuming and it is not always possible to recover the entire cost. We are aware that the study has limitations, among which is the criterion used to divide the size of hospitals. As will be verified in future studies, it is possible to divide a hospital into operating revenue and the value of its total assets. The validity of the research could also be improved with a full sample of poviat-commune and Marshal hospitals, and it would be worth trying to broaden the scope of the study to include University and Ministry hospitals. This would give an overview of all types of hospitals and their impact on the dependence on the founding authorities.

## 5. Conclusions

The healthcare system debt is a complex and volatile phenomenon. This is related to the need for hospitals to adapt to the changing local environment, regional disproportions between demand and supply in medical services, an imbalance in the allocation of funds to individual regions, and the excessive spatial concentration of health care facilities.

The difficult financial situation of hospitals, manifested mainly in negative financial results and systematic indebtedness of hospitals, is the main problem of the Polish healthcare system. Despite two attempts to reduce the debt of hospitals, the situation has still not improved. It seems that the sources of the difficult financial situation can be seen both in the ineffectiveness of the healthcare system and in the improper management of hospitals by their owners and managers. This problem was confirmed by the analysis of the literature on the subject.

Public hospitals in Poland can be established in various legal forms by a few legally specified entities. One of such entities are local government units. They have a significant impact on the proper functioning of the entire health care system in Poland. After the reform in 1999, they took over responsibility for the functioning of healthcare at the local and regional level. Thus, they began to perform ownership functions in relation to public hospitals. They make decisions related to the establishment and liquidation of hospitals. However, they are primarily responsible for their financial management.

The analysis carried out in the article leads to the conclusion that the financial situation of Polish public hospitals is diverse. This was also confirmed in the literature review [54]. As the statistical analysis showed, the vast majority of public hospitals are indebted. As shown in the Results section, comparing the average values of the DT and SLV variables according to ownership structure, in the group of medium-sized hospitals, it turned out that Marshal hospitals had better values (closer to the recommended values) than poviat-commune ones and hence were less indebted. In the case of large hospitals, the situation was reversed. The formation of solvency and debt ratios indicates problems in the operation of public hospitals in Poland, which is consistent with existing analyses [5,31]. The optimum values of the solvency ratio suggested by the Polish Ministry of Health should range between 0.01 and 0.5.

Nevertheless, a substantial minority of hospitals did not achieve these optimum values; instead, they reported negative ones. Negative solvency ratio values were associated with negative equity capital values affected by the balance sheet item: a loss brought forward or inefficiently using external financing. The debt ratios of the analyzed hospitals significantly exceeded the 0.4 level recommended by the Ministry of Health. The vast majority of hospitals showed values above 1.00, undermining their credibility. The number of hospitals meeting the recommended indebtedness level fell between 2013 and 2017.

The obtained results indicate the lack of a statistically significant influence of both the size and the founding body on the financial condition of the hospital. However, as shown by previous studies [3], there is considerable variation in the financial and organizational performance of hospitals. Therefore, one should consider the causes of their occurrence. Perhaps they result from the scope of medical services offered or the terms of contracting services with the National Health Fund. This could explain the direct impact on the diversified income and cost structure of these hospitals [10]. Moreover, as recent policy actions show, there have been calls to increase the role of voivodeships in coordinating the healthcare activities of the lower levels of territorial self-government, due to difficulties in local-government coordination [55]. Based on the conducted study and literature research, it should be stated that organizational changes integrated into a well-thought-out hospital strategy (using appropriate controlling tools) [56] seem to be a good way to improve financial results and achieve profitability at the level of core operations. Even though the hypotheses have not been verified positively, one should not underestimate the problematic situation of the healthcare sector in Poland. It should be underlined that supporting the financial sustainability of the hospital sector has become essential and will be increasingly important. Nowadays, hospitals face a challenging situation related to the COVID-19 pandemic. As Dubas–Jakóbczyk [14] showed, in 2020, hospitals were at the frontline of the fight against the pandemic, and they face huge pressures. Therefore, the need to analyze the financial situation and determine the various factors is vital.

The added value of our research is the analysis of the situation of public hospitals in Poland in terms of their financial performance, according to size and ownership group. The study fills the gap in comparative studies of the financial performance of public hospitals. Taking this into consideration, the authors believe that the findings of the study contribute to the literature on the financial performance of public hospitals in Poland. We believe that the article opens the field for further discussion on possible reasons for differentiating the financial situation depending on the founding body.

## Figures and Tables

**Table 1:** Verification of χ^2^ test of homogeneity.

Values (in %)	FB_U	FB_M	FB_MIN	FB_PC	Sum
Observed (*O_j_*)	10.0	41.7	10.0	38.3	100
Expected (*E_j_*)	7.0	36.0	8.0	49.0	100
χ^2^ = 7.815					

Note: significance level α = 0.05; FB_U, university hospital; FB_M, Marshal hospital; FB_MIN, ministry hospital; FB_PC, poviat-commune hospital. Source: own elaboration.

**Table 2:** List of variables used in the financial and economic analysis of healthcare.

Variable	Author, Year
Indebtedness (debt ratio, debt-to-equity ratio, and solvency ratio)	[5,10,14,27,28,30,31,32,33,34,35]
Ownership (founding body) and status (public/private unit)	[10,24,26,27,31,36,37,38,39]
Size of the hospital (hospital beds)	[4,9,14,18,26,31,38,40,41,42]

**Table 3:** Debt and solvency ratios—calculation methods.

Variable	Calculation Method
Debt ratio	$\frac{\mathrm{Liabilities}\;\mathrm{and}\;\mathrm{provisions}\;\mathrm{for}\;\mathrm{liabilities}}{\mathrm{Balance}\;\mathrm{sheet}\;\mathrm{total}}$
Solvency ratio	$\frac{Ll+Cl+Acc+Sf}{EC}$

Notes: Ll, long-term liabilities; Cl, current liabilities; Acc, accruals; Sf, special funds; Pl, provisions for liabilities; EC, equity capital; liabilities and provisions for liabilities = Ll + Cl + Pl + SF + Acc.

**Table 4:** Descriptive statistics of analyzed groups of hospitals.

Types of Hospitals/Variables										
Medium Marshal hospitals										
	2017.DT	2016.DT	2015.DT	2014.DT	2013.DT	2017. SLV	2016. SLV	2015. SLV	2014. SLV	2013. SLV
Mean	0.77	0.76	0.78	0.75	0.76	−3.15	−7.70	1.40	−16.78	2.19
Median	0.72	0.71	0.72	0.71	0.71	0.84	0.93	0.89	0.88	1.02
Stand. Dev.	0.39	0.39	0.39	0.39	0.39	42.52	81.47	9.55	160.11	9.10
Large Marshal hospitals										
	2017.DT	2016.DT	2015.DT	2014.DT	2013.DT	2017. SLV	2016. SLV	2015. SLV	2014. SLV	2013. SLV
Mean	0.68	0.67	0.68	0.66	0.64	1.75	2.48	1.02	3.94	2.37
Median	0.62	0.61	0.62	0.60	0.60	1.01	0.95	1.10	0.98	1.01
Stand. Dev.	0.34	0.34	0.34	0.35	0.32	11.20	14.46	9.43	22.76	15.97
Medium poviat-commune hospitals										
	2017.DT	2016.DT	2015.DT	2014.DT	2013.DT	2017. SLV	2016. SLV	2015. SLV	2014. SLV	2013. SLV
Mean	0.75	0.75	0.76	0.74	0.76	−0.49	−2.27	1.30	−5.85	−4.15
Median	0.71	0.71	0.72	0.69	0.69	0.77	0.79	0.77	0.78	0.60
Stand. Dev.	0.42	0.42	0.42	0.42	0.51	22.34	31.27	20.54	56.06	37.93
Large poviat-commune hospitals										
	2017.DT	2016.DT	2015.DT	2014.DT	2013.DT	2017.SLV	2016.SLV	2015.SLV	2014.SLV	2013.SLV
Mean	0.82	0.81	0.82	0.80	0.79	1.93	3.69	0.17	7.22	−0.07
Median	0.78	0.78	0.77	0.78	0.77	1.41	1.40	1.55	1.37	1.01
Stand. Dev.	0.38	0.39	0.38	0.40	0.38	7.25	11.00	15.81	32.49	7.95

Stand. Dev., standard deviation. Source: own calculations based on data provided by the Amadeus and EMIS databases.

**Table 5:** Results of the Mann–Whitney U test for SLV in medium-sized hospitals.

	Medium-Sized		Large	
Variable	Z	*p*-Value	Z	*p*-Value
2017	0.507936	0.611498	−0.64873	0.516513
2016	0.489632	0.624394	−1.04662	0.295277
2015	0.453024	0.650531	−0.66026	0.509085
2014	0.288288	0.773126	−0.89669	0.369885
2013	1.290433	0.196901	0.10668	0.915043

Source: own calculations based on data provided by Amadeus and EMIS databases.

**Table 6:** Results of the Mann–Whitney U test for SLV for Marshal hospitals.

	Marshal Hospitals		Poviat-Commune Hospitals	
Variable	Z	*p*-Value	Z	*p*-Value
2017	−0.247110	0.804823	0.760620	0.446885
2016	−0.223798	0.822915	1.269585	0.204234
2015	−0.293735	0.768961	0.788896	0.430174
2014	0.083924	0.933117	1.167792	0.242892
2013	−0.153861	0.877719	0.641861	0.520964

Source: own calculations based on data provided by Amadeus and EMIS databases.

## Data Availability

The data presented in this study are available on request from the corresponding author.
